# Automation of [^18^F]fluoroacetaldehyde synthesis: application to a recombinant human interleukin‐1 receptor antagonist (rhIL‐1RA)

**DOI:** 10.1002/jlcr.3393

**Published:** 2016-04-06

**Authors:** Olivia Morris, Adam McMahon, Herve Boutin, Julian Grigg, Christian Prenant

**Affiliations:** ^1^ Wolfson Molecular Imaging Centre, CRUK and EPSRC Cancer Imaging Centre in Cambridge and ManchesterThe University of ManchesterManchesterUK; ^2^ GE Healthcare, Life Sciences, Imaging R&DThe Grove CentreAmershamBucksUK

**Keywords:** [^18^F]fluoroacetaldehyde, [^18^F]rhIL1RA, protein radiolabelling, prosthetic group, PET

## Abstract

[^18^F]Fluoroacetaldehyde is a biocompatible prosthetic group that has been implemented pre‐clinically using a semi‐automated remotely controlled system. Automation of radiosyntheses permits use of higher levels of [^18^F]fluoride whilst minimising radiochemist exposure and enhancing reproducibility. In order to achieve full‐automation of [^18^F]fluoroacetaldehyde peptide radiolabelling, a customised GE Tracerlab FX‐FN with fully programmed automated synthesis was developed.

The automated synthesis of [^18^F]fluoroacetaldehyde is carried out using a commercially available precursor, with reproducible yields of 26% ± 3 (decay‐corrected, *n* = 10) within 45 min. Fully automated radiolabelling of a protein, recombinant human interleukin‐1 receptor antagonist (rhIL‐1RA), with [^18^F]fluoroacetaldehyde was achieved within 2 h. Radiolabelling efficiency of rhIL‐1RA with [^18^F]fluoroacetaldehyde was confirmed using HPLC and reached 20% ± 10 (*n* = 5).

Overall RCY of [^18^F]rhIL‐1RA was 5% ± 2 (decay‐corrected, *n* = 5) within 2 h starting from 35 to 40 GBq of [^18^F]fluoride. Specific activity measurements of 8.11–13.5 GBq/µmol were attained (*n* = 5), a near three‐fold improvement of those achieved using the semi‐automated approach.

The strategy can be applied to radiolabelling a range of peptides and proteins with [^18^F]fluoroacetaldehyde analogous to other aldehyde‐bearing prosthetic groups, yet automation of the method provides reproducibility thereby aiding translation to Good Manufacturing Practice manufacture and the transformation from pre‐clinical to clinical production.

## Introduction

Positron emission tomography (PET) is an imaging modality permitting quantitative non‐invasive *in‐vivo* molecular imaging; its increasing popularity in a clinical setting is attributable to its sensitivity and ability to identify unique biomarkers of disease and capacity to classify physiological processes at the molecular level. The ability to automate radiotracer synthesis has been key in the advancement of PET.^1^ It not only improves reproducibility which eases transition to Good Manufacturing Practice (GMP) manufacture, a key objective of novel radiotracer development, but also allows GBq scale quantities of [^18^F]fluoride to be used with minimal radiochemist exposure. The GE TRACERLab FX series of synthesisers are commonly used as radiochemistry platforms, and a customised FX‐FN system has been used to automate the [^18^F]fluoroacetaldehyde synthesis originally described by Prenant *et al.* 2, 3 which used a semi‐automated experimental setup.

[^18^F]Fluoride is a PET radioisotope with a number of advantageous features including 97% positron emission, a low positron energy and a 109.8‐min radioactive half‐life, which permits more extensive radiochemical syntheses yet also limits patient dose.^4^, ^5^

Peptides and proteins are complex and have multiple functionalities that are incompatible with direct fluorination using [^18^F]fluoride. It is for this reason that prosthetic groups capable of being covalently linked to the peptide under mild reaction conditions are used. [^18^F]Fluoroacetaldehyde is an important prosthetic group owing to its small size and, importantly, its solubility in water thereby removing the requirement for organic solvents that might denature a protein or peptide. The volatility of fluoroacetaldehyde permits purification via distillation, eliminating the requirement for solid phase extraction (SPE) or high performance liquid chromatography (HPLC) separation.^3^ To date, [^18^F]fluoroacetaldehyde has been successfully implemented in a pre‐clinical trial using [^18^F]fluoroacetaldehyde radiolabelled recombinant human interleukin‐1 receptor antagonist (rhIL‐1RA), 17.5 kDa.^2^ Radiosynthesis of [^18^F]fluoroacetaldehyde from [^18^F]fluoride achieved radiochemical yields (RCY) of 31–37% in a two‐step, one‐pot reaction scheme.^3^ This was achieved in a semi‐automated synthesis using a remotely controlled experimental system and reached overall RCY of [^18^F]rhIL‐1RA ranging from 7.1 to 24.2%.^2^

Here, we describe the fully‐automated synthesis of [^18^F]fluoroacetaldehyde and subsequent radiolabelling of rhIL‐1RA using a modified GE TRACERLab FX‐FN. Radiolabelling of rhIL‐1RA with [^18^F]fluoroacetaldehyde permits direct comparison between the methods of Prenant *et al.* 2 and the present study.

**Figure 1:**
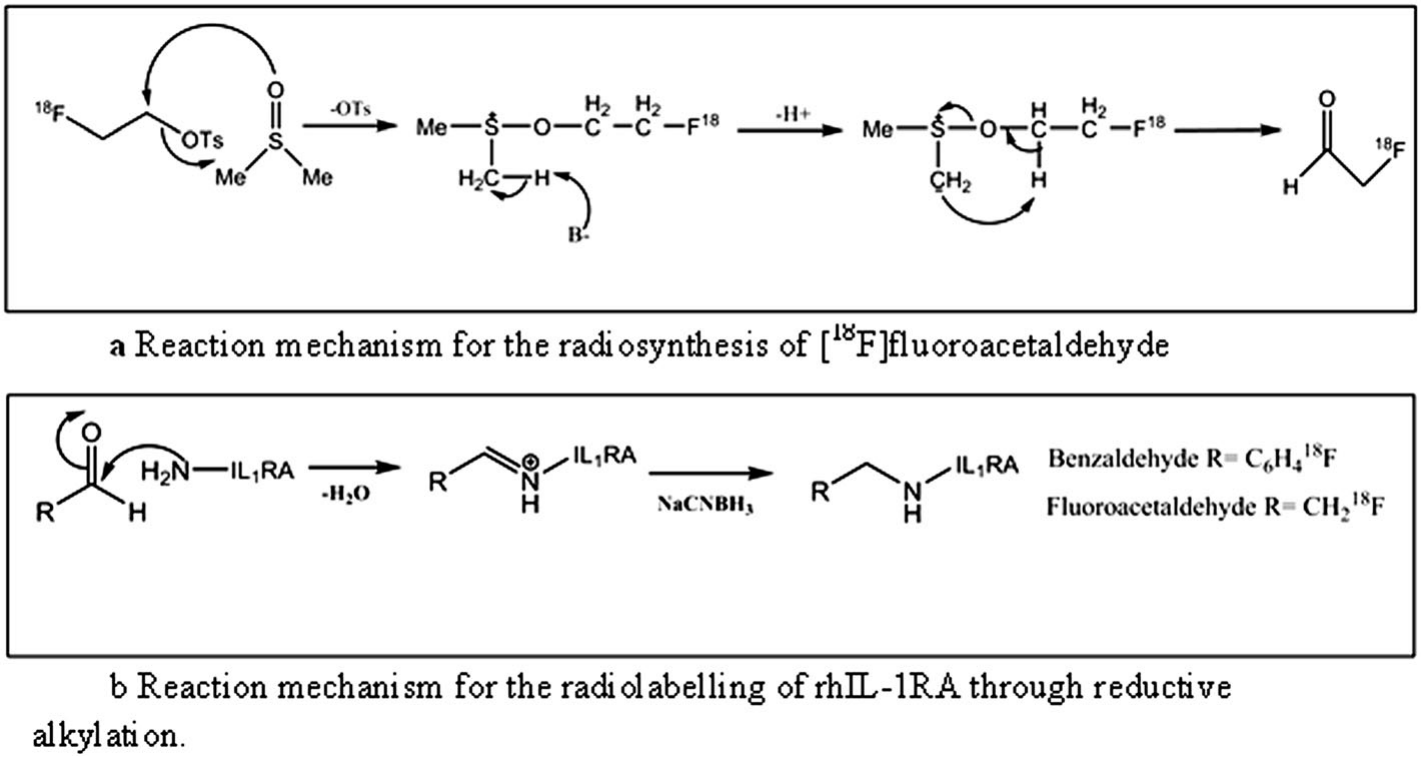
(a) Reaction mechanism for the radiosynthesis of [^18^F]fluoroacetaldehyde. (b) Reaction mechanism for the radiolabelling of rhIL‐1RA through reductive alkylation.

Figure 1a shows the reaction mechanism for the synthesis of [^18^F]fluoroacetaldehyde, whilst Figure 1b illustrates the mechanism of rhIL‐1RA reductive alkylation with [^18^F]fluoroacetaldehyde.

## Experimental

All solvents were purchased from Sigma‐Aldrich (Gillingham, Dorset, UK) and used without further purification. rhIL‐1RA (100 mg/ml), anakinra (Kineret) was provided by Amgen (Thousand Oaks, CA, USA) in a formulated solution containing 100‐mg anakinra, 1.29‐mg sodium citrate, 5.48‐mg sodium chloride, 0.12‐mg disodium EDTA and 0.70‐mg polysorbate 80 in 1‐ml water.

[^18^F]Fluoride was produced onsite via the ^18^O(*p*, *n*)^18^F nuclear reaction by 16.4‐MeV proton bombardment of enriched [^18^O]H_2_O using a GE (Amersham, Bucks, UK) PETtrace cyclotron.

Analytical HPLC was performed using a Shimadzu (Milton Keynes, UK) Prominence system (LC‐20AB solvent delivery system, SPD‐20A dual wavelength absorbance detector) controlled by LabLogic (Sheffield, UK) Laura 3 software via a CBM‐20A controller. HPLC eluate was measured for radioactivity using a Bioscan (Oxford, UK) Flowcount B‐FC 3100 gamma detector. All pre‐clinical PET scans were carried out using a Siemens (Oxford, UK) Inveon® PET‐CT scanner.

## Radiosynthesis, GE TRACERLab FX‐FN

### [^18^F]Potassium fluoride

**Figure 2:**
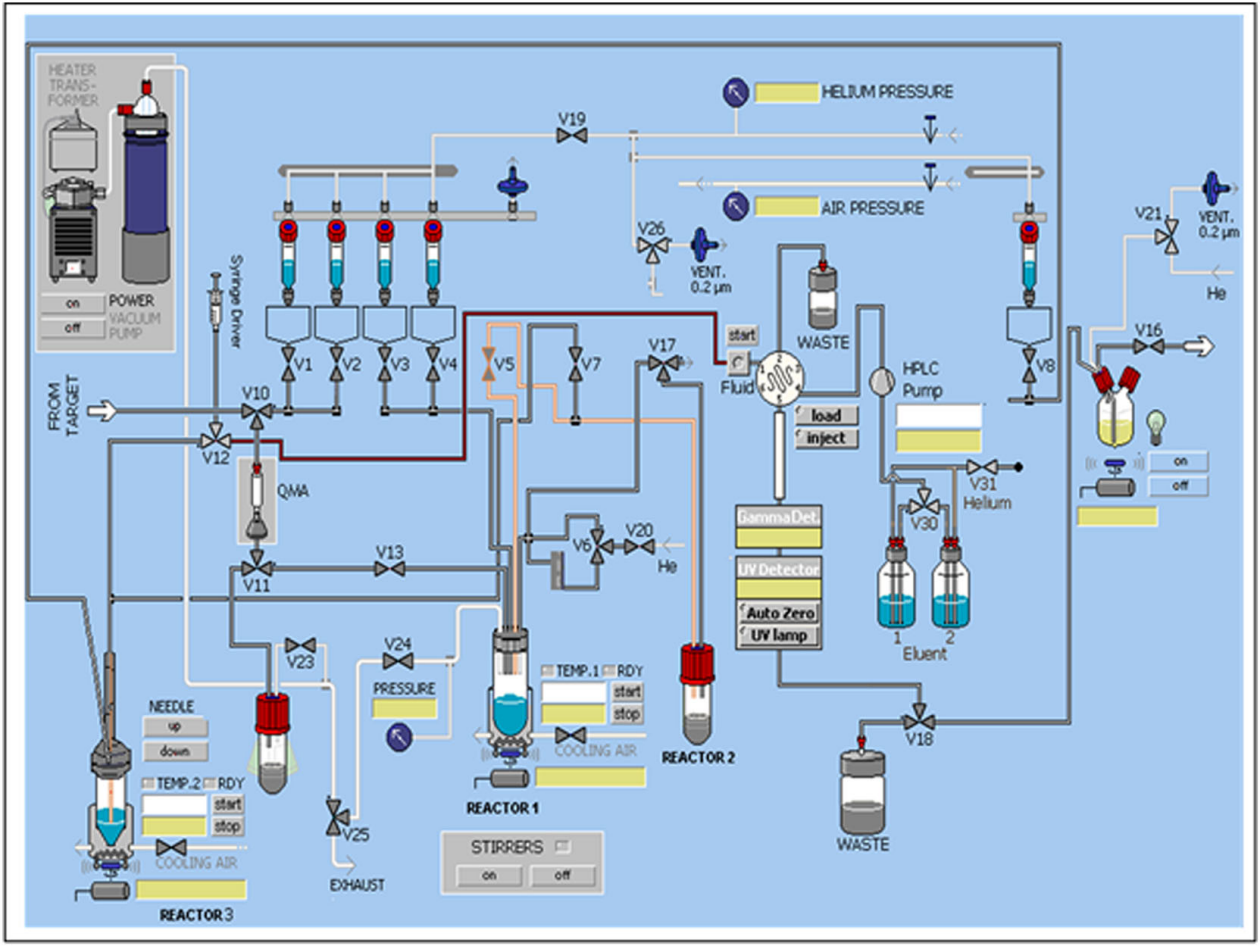
Schematic of GE TRACERlab FX‐FN setup.

Cyclotron produced [^18^F]Fluoride was trapped on a Sep‐Pak QMA cartridge (Oasis, Waters, Wilmslow, UK) then eluted with K_2_CO_3_ solution (4 µmol, 0.4 ml) into a 3‐ml vial (Reactor 1 on Figure 2) containing Kryptofix 222 (3 mg, 8 µmol) in acetonitrile (0.7 ml). The mixture was azeotropically dried at 110 °C with 3 sequential additions of acetonitrile (3 × 0.5 ml).

### [^18^F]Fluoroethyltoslyate (FEtTos)

A solution of ethylene di‐*p*‐toluenesulphonate (5 mg, 13.5 µmol) in DMSO (150 µl) was added from Vial 3 (V3 on Figure 2) to dried [^18^F]KF/Kryptofix complex in Reactor 1. The reactor was then heated to 80 °C and cooled to 50 °C after 10 min HPLC analysis of the crude reaction mixture was performed to measure [^18^F]FEtTos RCY using a Prodigy column (10 µm, ODS (3), 100 Å. 250 × 10 mm Phenomenex UK, Macclesfield, Cheshire UK) eluted with acetonitrile:water 50:50, 6 ml/min, 254 nm, *t*_R_ = 9 min.

### [^18^F]Fluoroacetaldehyde

To the reactor containing [^18^F]FEtTos in DMSO was added DMSO (150 µl) from vial 4 (V4 on Figure 2). The reactor was then heated to 160 °C for 5 min, and a route from Reactor 1 to Reactor 2 was opened (through V5). [^18^F]Fluoroacetaldehyde was distilled and collected in Reactor 2 containing pH 6 citrate buffer (150 µl, 20 mM). Distillation proceeded for 4 min to minimise DMSO carry‐over. RCY 26% ± 3 (decay‐corrected, *n* = 10).

### [^18^F]rhIL‐1RA

[^18^F]Fluoroacetaldehyde was transferred from Reactor 2 to Reactor 3 (see Figure 2) containing a solution of rhIL‐1RA (30 µl, 1.7 µmol), sodium cyanoborohydride in a solution of pH 6 citrate buffer (10 µl, 1.0 M) and pH 6 citrate buffer (55 µl, 20 mM) before heating to 40 °C for 20 min. [^18^F]Fluoroacetaldehyde radiolabelled rhIL‐1RA was purified using SE‐HPLC (GE Superdex 200 10/300 GL, PBS, 1 ml/min, 254 and 220 nm, *t*_R_ = 15 min) and analysed for quality control purposes using analytical SE‐HPLC (GE Superdex 200 10/300 GL, PBS eluent, 0.6 ml/min, 254 and 220 nm, *t*_R_ = 15 min). Overall RCY and specific activity measurements of 5% ± 2 and 8.11–13.5 GBq/µmol respectively (decay‐corrected, *n* = 5). Specific activity measurements were calculated using a standard of rhIL‐1RA as a reference for HPLC analysis.

### [^18^F]fluoroacetaldehyde‐2,4‐DNPH

Derivatisation of [^18^F]fluoroacetaldehyde was performed using the methodology reported by Prenant *et al*.^3^ In short, 2,4‐dinitrophenylhydrazine (DNPH) (25 µl, 5 µmol) was added to [^18^F]fluoroacetaldehyde (30 µl) in ethanol (60 µl). The solution was heated for 15 min at 90 °C before HPLC analysis (Phenomenex Prodigy C18, 10 µm, ODS (3), 100 Å. 250 × 10 mm, acetonitrile:water 50:50, 3 ml/min, 360 nm, *t*_R_ = 25 min).

## Synthesis of reference compounds

### Synthesis of fluoroacetaldehyde

Fluoroacetaldehyde was prepared as per the methodology reported by Prenant *et al*.^3^ In short, fluoroethanol (62 µl, 1 mmol) was added to pyridinium dichromate (PDC) (576 mg, 1.5 mmol) in dichloromethane (DCM) (1.5 ml) and was stirred at room temperature for 24 h. The liquid portion was removed from the PDC precipitate and then heated to 80 °C. The distillate of which was collected in deionised H_2_O (1 ml), and a sample of aqueous phase, containing the fluoroacetaldehyde, was used for HPLC analysis (Phenomenex Prodigy C18, 10 µm, ODS (3), 100 Å. 250 × 10 mm, water eluent, 2 ml/min, 277 nm, *t*_R_ = 8 min).

### Synthesis of fluoroacetaldehyde‐2,4‐DNPH

To the fluoroacetaldehyde solution (0.5 ml) 2,4‐DNPH phosphoric acid solution (0.8 ml, 0.2 M) was added, and the mixture was heated to 90 °C for 15 min before HPLC analysis (Phenomenex Prodigy C18, 10 µm, ODS (3), 100 Å. 250 × 10 mm, acetonitrile:water 50:50, 3 ml/min, 360 nm, *t*_R_ = 25 min).

### Pre‐clinical PET imaging

All animal handling was in accordance with UK legislation under the 1986 Animals (Scientific Procedures) Act. The pre‐clinical methodology followed was as described by Cawthorne *et al.* in 2011.^6^ In short, one C57Bl6 mouse and one Sprague–Dawley rat were anaesthetised using isoflurane (induction 4% and maintained 1.5%) in 70% N_2_O and 30% O_2_ mixture. [^18^F]rhIL‐1RA was injected in the tail vein (17.9 MBq for the mouse and 21.4 MBq for the rat). The scans were performed on a Siemens Inveon® PET‐CT scanner. The acquisition protocol consisted of the following parameters: a CT scan was performed prior to the PET acquisition to obtain the attenuation correction factors. PET data were acquired over 1 h with the time coincidence window set to 3.432 ns and the levels of energy discrimination set to 350 keV and 650 keV. The list mode acquisition data files were histogrammed into 3D sinograms with a maximum ring difference of 79 and span 3. The list mode data of the emission scans were sorted into 16 dynamic frames (5 × 1 min, 5 × 2 min, 3 × 5 min, 3 × 10 min). Finally, the emission sinograms (each frame) were normalised, corrected for attenuation, scattering and radioactivity decay, and reconstructed using OSEM3D (16 subsets and 4 iterations) into images of dimensions 128^2^ (transaxially) × 159 (longitudinally) with 0.776 × 0.776 × 0.796 mm voxels (FOV diameter: 99.3 mm × 126.6 mm longitudinally).

## Results and discussion

A schematic of the customised GE TRACERlab FX‐FN can be seen in Figure 2. The modifications permitting automated [^18^F]fluoroacetaldehyde synthesis and subsequent rhIL‐1RA radiolabelling will now be discussed alongside details regarding process development and finally [^18^F]rhIL‐1RA pre‐clinical results.

### Addition of a third reactor

A third reactor was added to the GE TracerLab FX‐FN in order to mitigate issues of detrimental peptide foaming upon prolonged exposure to a gas flow. The process of [^18^F]fluoroacetaldehyde distillation requires constant helium flow at 15 ml/min and, in order to collect the volatile product, it is necessary to distill the [^18^F]fluoroacetaldehyde into a solution; it was found that this process caused excessive foaming of the peptide and prevented a high‐yielding reaction. In the current setup, [^18^F]fluoroacetaldehyde is distilled into citrate buffer pH 6 (150 µl), in a second reactor, and this solution is then transferred to a third reactor. The ability to both retract and insert the needle in to and out of the solution in Reactor 3 was an important modification. In the first instance, addition of the [^18^F]fluoroacetaldehyde/buffer solution whilst the needle is in a retracted position ensures minimal exposure of the peptide to helium flow. Once the transfer of [^18^F]fluoroacetaldehyde to Reactor 2 is complete, the needle can be lowered into the reaction mixture to be transferred to the HPLC loop and injected onto the HPLC column. This, therefore, required the reactor head to be moved from its original position at Reactor 1 to Reactor 3. A buffer volume of 150 µl in Reactor 2 was required to minimise the degree of loss during the transition from Reactor 2 to 3. As a result, the peptide reaction mixture volume in Reactor 3 was kept to a minimum to maintain a high concentration for radiolabelling.

### Distillation reaction conditions

Distillation was carried out using Reactors 1 and 2. As the reactor head had to be moved from Reactor 1 to Reactor 3, it was necessary to produce a home‐made reactor, with silicon septum screw top, that fitted directly into the heating block, as can be seen in Figure 2.

A temperature of 160 °C was required for distillation of [^18^F]fluoroacetaldehyde from Reactor 1 to 2. This temperature achieved a reproducible RCY of the prosthetic group and minimised the degree of DMSO carry‐over into the second reactor. It was established that an increase in reactor 1 temperature from 160 °C to 170 °C had minimal impact on the RCY of [^18^F]fluoroacetaldehyde and increased the amount of DMSO carry‐over into Reactor 2, an undesirable result owing to the sensitivity of some peptides and proteins to DMSO.

A distillation time of 4 min was sufficient to reach a plateau in the RCY of [^18^F]fluoroacetaldehyde. Additional time resulted in further DMSO carry‐over.

Prenant *et al.* 2 describe a flow‐rate of 7–8 ml/min in the reductive alkylation of rhIL‐1RA with [^18^F]fluoroacetaldehyde. Therefore a flow‐meter and needle‐valve, connected to valve 6, were incorporated in the automated setup to control the helium flow for the distillation step between Reactor 1 and Reactor 2. A flow‐rate of 15 ml/min was found to produce the optimal distillation conditions.

[^18^F]Fluoroacetaldehyde syntheses reproducibly attained RCY of 26% ± 3 (decay‐corrected, *n* = 10) within 45 min starting from 35 to 40 GBq of [^18^F]fluoride.

### Synthesis of [^18^F]FEtTos in DMSO to remove acetonitrile evaporation step

**Figure 3:**
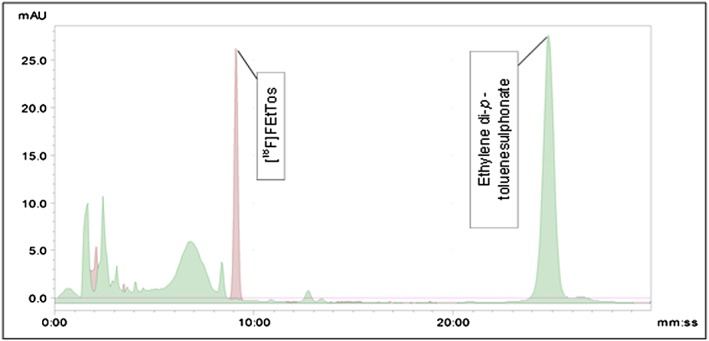
Radio‐ and UV‐chromatogram for the radiosynthesis of [^18^F]FEtTos in DMSO.

Block *et al.* 7 report synthesising [^18^F]FEtTos in acetonitrile, achieving appreciable RCY >90% within 10 min. The synthesis of [^18^F]fluoroacetaldehyde requires Kornblum oxidation thus requiring DMSO. As a result of this and for ease of automation, the synthesis of [^18^F]FEtTos in DMSO was investigated. Figure 3 gives the radio‐UV‐chromatogram data from the synthesis of [^18^F]FEtTos in DMSO, achieving reproducible RCY of 60%. As a consequence of this, DMSO was used for the synthesis of [^18^]FEtTos. [^18^F]FEtTos can be seen at 9 min, and the ditosylate precursor can be seen in the UV chromatogram at 25 min.

It should be noted that, despite DMSO being used to carry out [^18^F]FEtTos synthesis, an additional DMSO aliquot was required for the Kornblum oxidation and production of [^18^F] fluoroacetaldehyde. This may be attributable to degradation of the initial DMSO aliquot used during [^18^F]FEtTos synthesis.

### [^18^F]Fluoroacetaldehyde characterisation with 2,4‐DNPH

The synthesis of [^18^F]fluoroacetaldehyde was confirmed by derivatisation to the corresponding hydrazone followed by radio‐HPLC analysis. 2,4‐DNPH was used as the derivatising agent.^8^

**Figure 4:**
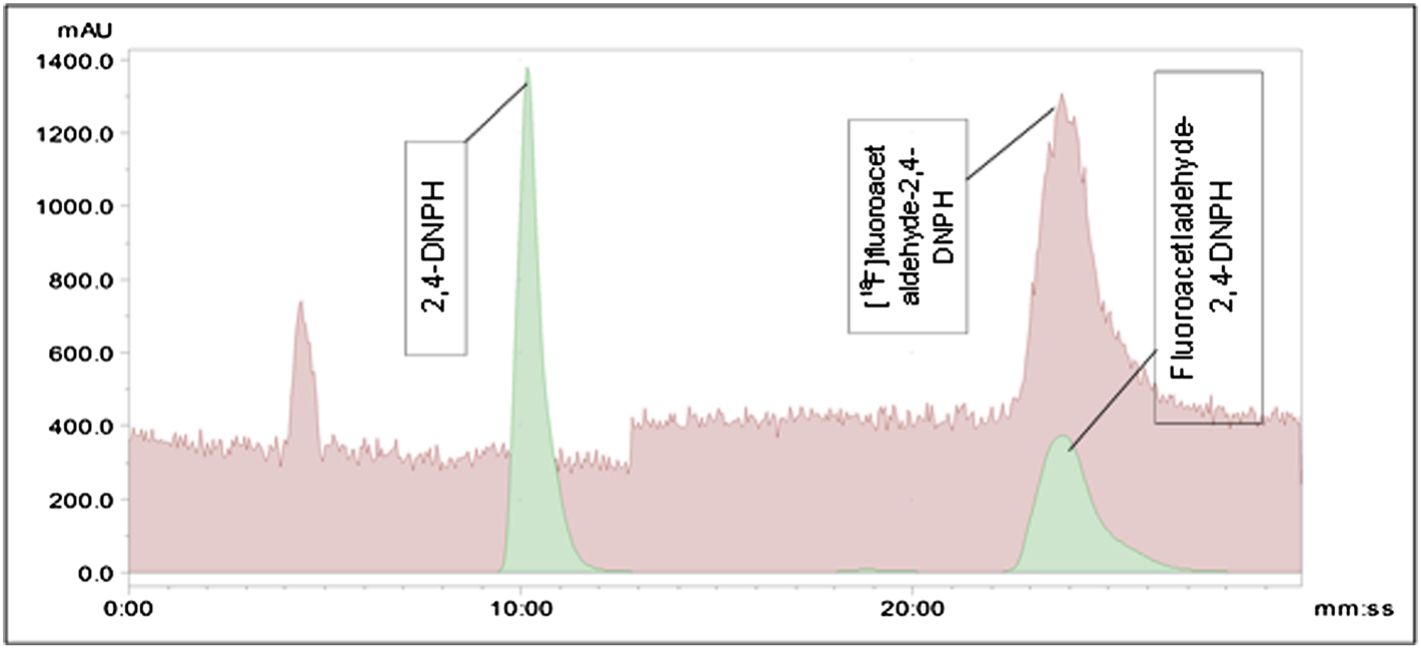
Radio‐ and UV‐chromatogram for the characterisation of [^18^F]fluoroacetaldehyde with 2,4‐DNPH.

Figure 4 gives the radio‐UV‐chromogratogram data from a co‐injection of isotopically unmodified‐fluoroacetladehyde‐2,4‐DNPH solution and [^18^F]fluoroacetaldehyde‐2,4‐DNPH solution. 2,4‐DNPH has a retention time of approx. 10 min and co‐elution of the corresponding hydrazone complex for both [^18^F]fluoroacetaldehyde and isotopically unmodified fluoroacetaldehyde is seen at approximately 24 min.

### Radiolabelling of rhIL‐1RA with [^18^F]fluoroacetaldehyde

**Figure 5:**
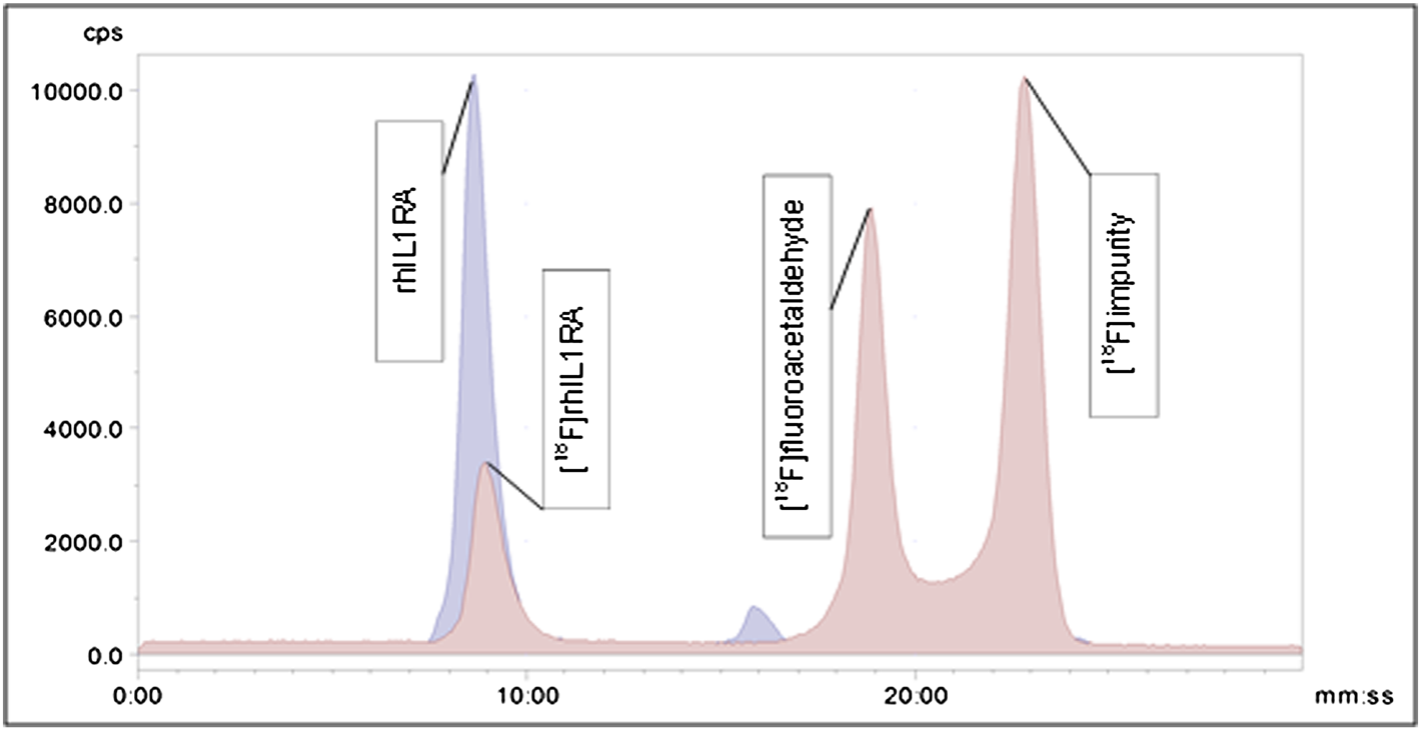
Crude reaction mixture of [^18^F]fluoroacetaldehyde and rhIL‐1RA.

Radiolabelling of rhIL‐1RA with [^18^F]fluoroacetaldehyde was carried out at 40 °C for 20 min. Radiolabelling efficiency of rhIL‐1RA with [^18^F]fluoroacetaldehyde was confirmed using HPLC and reached 20% ± 10 (*n* = 5). The radio‐ and UV‐chromatogram can be seen in Figure 5.

The SE‐HPLC chromatogram shows the [^18^F]rhIL‐1RA at 9 min and [^18^F]fluoroacetaldehyde at 19 min. A side‐product is also observed at 23 min, thought to be as a result of a competing reaction with the reducing agent. This would be resolved under chemical‐ligation conditions on account of the enhanced stability of an oxime or hydrazide which negates the requirement of a reducing agent.

The overall RCY of [18 F]rhIL‐1RA was 5% ± 2 (decay‐corrected, *n* = 5) starting from 35 to 40 GBq of [^18^F]fluoride. Specific activity measurements of 8.11–13.5 GBq/µmol were attained (*n* = 5) using the automated method as opposed to 813.15–5040.2 MBq/µmol achieved using the semi‐automated approach.^2^ This significant increase in specific activity is a considerable advantage to the employ of the automated method and is a result of the ability to use higher starting levels of radioactivity (35–40 GBq compared to 1.73–3.42 GBq).

The relatively low RCY can be expected from reductive alkylation, on account of the concentration dependence of the second order rate kinetics and thus the relatively slow rates of reaction.^9^ The radiolabelling of a protein with [^18^F]fluoroacetaldehyde is not, however, limited to reductive alkylation, and the strategy can be used to site‐specifically radiolabel aminooxy‐ or hydrazide‐functionalised peptides or proteins. Additionally, the requirement to use larger reaction volumes in order to efficiently transfer reaction mixtures around the platform can negatively impact the RCY. Higher RCY were attained under reductive alkylation in the original publication by Prenant *et al.*; however, the process was not compatible for translation to an automated platform.^2^, ^3^ In the semi‐automated procedure, a sephadex gel was used to stabilise the rhIL‐1RA which is prone to foaming, as described.^2^ It was not possible to stabilise the protein in this manner using the automated platform on account of the following SE‐HPLC purification step. Despite the automated synthesis attaining lower overall RCY than the semi‐automated configuration, the enhanced reproducibility and considerable specific activity improvements are significant advantages to use of the automated method.^2^

**Figure 6:**
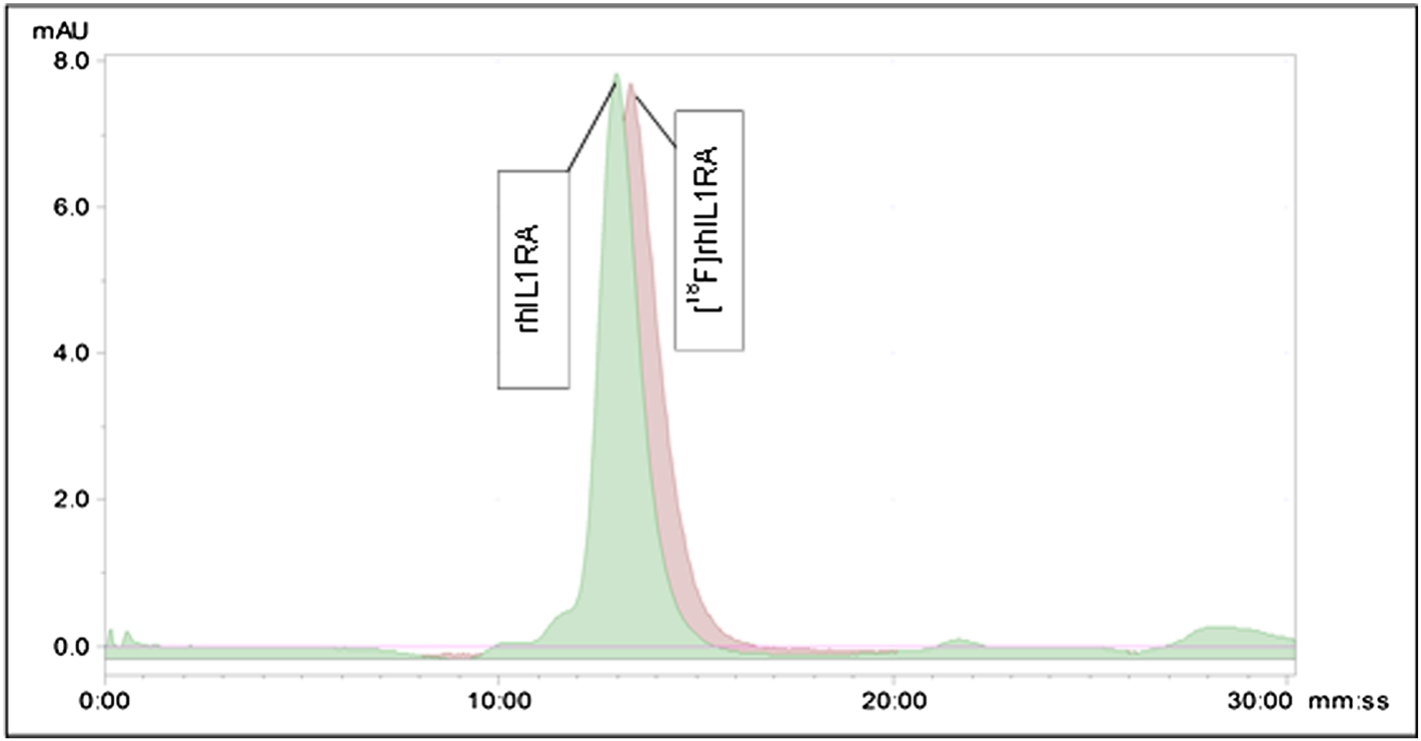
Quality control of [^18^F]fluoroacetaldehyde labelled rhIL‐1RA.

Importantly, [^18^F]fluoroacetaldehyde radiolabelling occurs in aqueous conditions and at low temperatures without the requirement for a high concentration of organic solvent, unlike other [^18^F]prosthetic groups such as [^18^F]fluorobenzaldehyde. Such conditions are likely to be more suitable for peptides and proteins. Figure 6 shows the quality control (QC) analysis of the formulated [^18^F]fluoroacetaldehyde labelled rhIL‐1RA. The SE‐HPLC chromatogram shows good radiochemical purity of the final radiolabelled product, although a small shoulder can be seen on the peak attributable to a dimer of the rhIL‐1RA. This was also reported by Prenant *et al.* in 2010.^2^

### Pre‐clinical results

**Figure 7:**
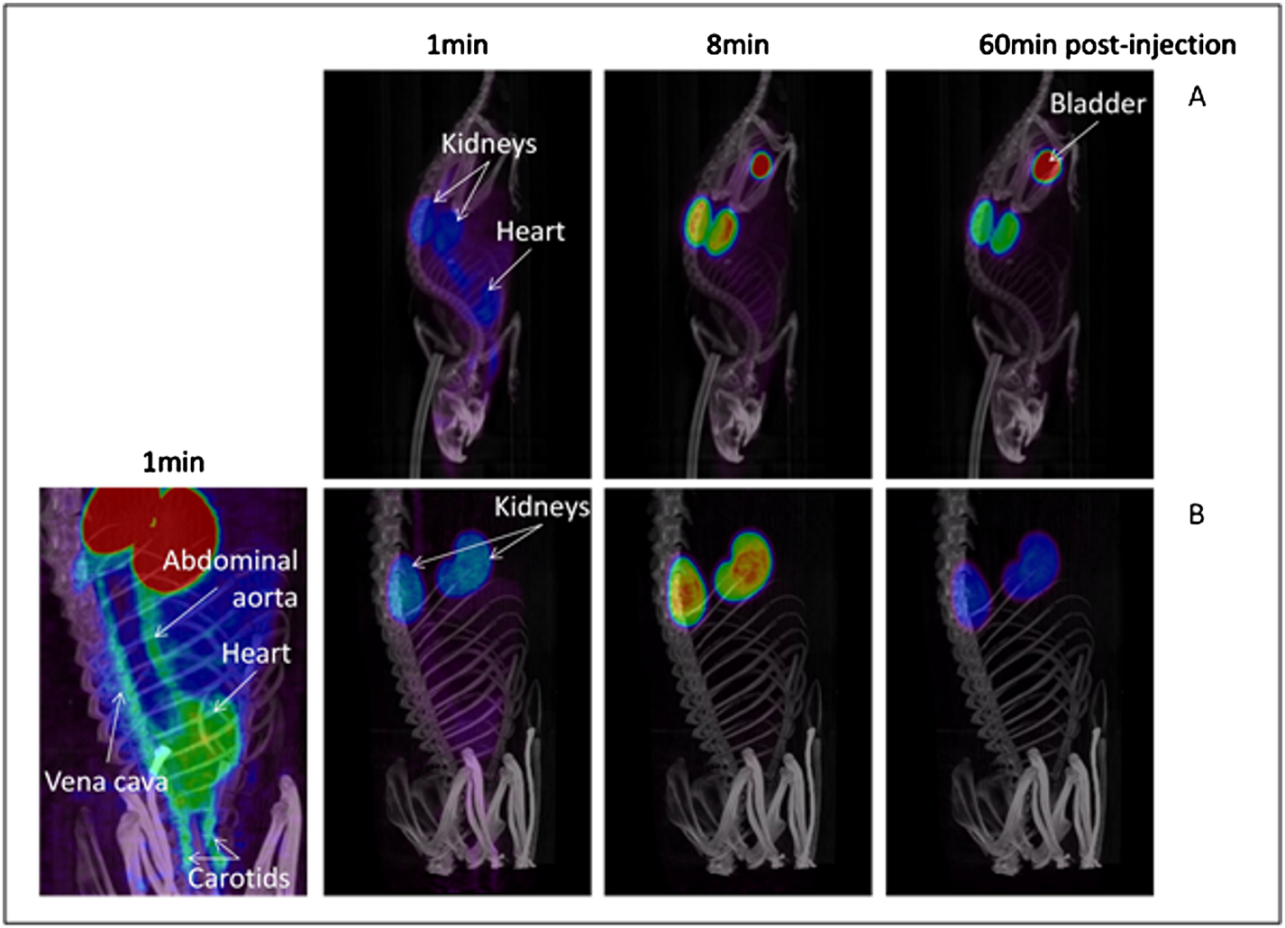
Mean intensity projection images of a mouse (A) and a rat (B) after injection of [^18^F]rhIL‐1RA.

^8^F]rhIL‐1RA was pre‐clinically assessed using both wild type rat and mouse models and can be seen in Figure 7. According to pre‐clinical analyses, the biodistribution shows no defluorination as would be evidenced by [^18^F]fluoride bone uptake. Preferential distribution of [^18^F]rhIL‐1RA in the kidneys and renal excretion was seen, as previously described by Cawthorne *et al.*,^6^ signifying preservation of rhIL‐1RA integrity.

The time activity curves (TAC) (see Supporting Information, Figure 1) show the concentration of [^18^F]rhIL‐1RA over time in the kidneys (cortex) and heart ventricle. The TAC presented show agreement between data acquired in the current work (shown in black) and the previous published work (shown in grey) which used a semi‐automated [^18^F]rhIL‐1RA radiosynthetic approach.^6^ The level quantified in the heart is lower than previously reported^6^ which is thought to be attributable to the absence of attenuation and scatter correction with the HIDAC scanner previously used when compared to the Inveon scanner used in the present study.

### Summary

We report upon an automated radiosynthesis of [^18^F]fluoroacetaldehyde and its successful application in radiolabelling rhIL‐1RA. [^18^F]Fluoroacetaldehyde syntheses reproducibly attained RCY of 26% ± 3 within 45 min (decay‐corrected, *n* = 10) starting from 35 to 40 GBq of [^18^F]fluoride. The automated platform additionally permitted successive radiolabelling of a protein with [^18^F]fluoroacetaldehyde. The 17.5‐kDa protein, rhIL‐1RA, was radiolabelled with [^18^F]fluoroacetaldehyde with labelling efficiencies of 20% ± 10 (*n* = 5), achieving overall RCY of 5% ± 2 within 2 h (decay‐corrected, *n* = 5) starting from 35 to 40 GBq of [^18^F]fluoride. Specific activity measurements of 8.11–13.5 GBq/µmol were achieved (*n* = 5). Use of the automated radiosynthesis of [^18^F]fluoroacetaldehyde and subsequent rhIL‐1RA labelling not only minimised radiochemist exposure, but importantly improved reproducibility and produced a marked improvement in specific activity measurements on account of the ability to start with higher levels of radioactivity.

## Disclosure statement

The authors declare no potential sources of conflict of interest or competing financial interest.

## Supporting information

Supporting info item
