# Supplementary figures and images for: Automation of [18F]fluoroacetaldehyde synthesis: application to a recombinant human interleukin‐1 receptor antagonist (rhIL‐1RA)
Source: J Labelled Comp Radiopharm. 2016 Apr 6;59(7):277–83. doi: 10.1002/jlcr.3393 (PMC4913750; doi:10.1002/jlcr.3393)

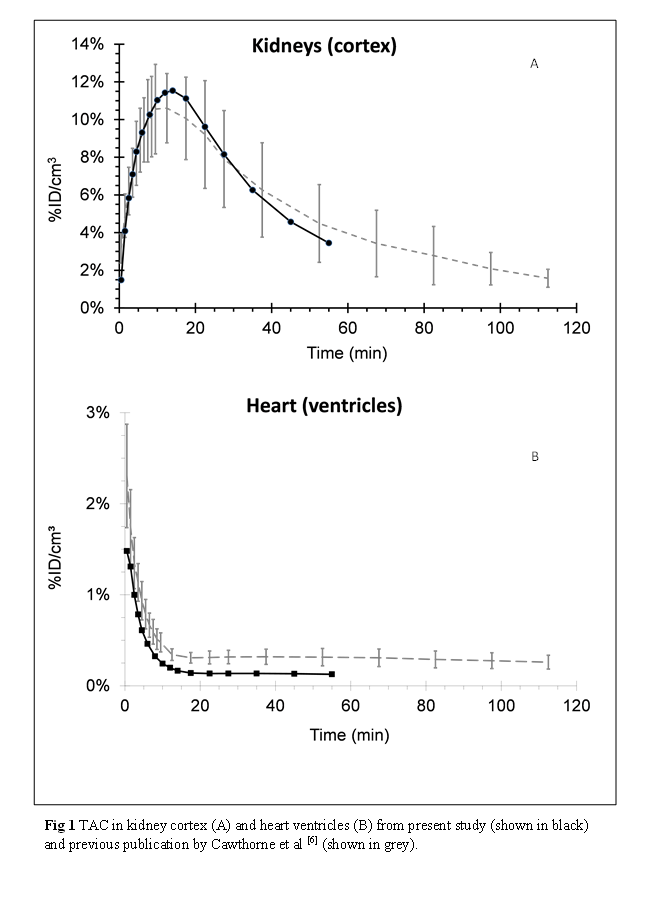

Supplement: Supplementary file 1 — Supporting info item [file JLCR-59-277-s001.tif]
